# Biomimetic lipid–fluorescein probe for cellular bioimaging

**DOI:** 10.3389/fchem.2023.1151526

**Published:** 2023-04-21

**Authors:** Hyungkyu Moon, Tania Sultana, JeongIk Lee, Jungrim Huh, Hae Dong Lee, Myung-Seok Choi

**Affiliations:** ^1^ Department of Materials Chemistry and Engineering, Konkuk University, Seoul, Republic of Korea; ^2^ Regenerative Medicine Laboratory, Center for Stem Cell Research, Department of Biomedical Science and Technology, Institute of Biomedical Science and Technology, Konkuk University, Seoul, Republic of Korea; ^3^ Department of Veterinary Obstetrics and Theriogenology, College of Veterinary Medicine, Konkuk University, Seoul, Republic of Korea; ^4^ Social Eco-Tech Research Institute, Konkuk University, Seoul, Republic of Korea

**Keywords:** fluorescein, phospholipid, fluorescent probe, bioimaging, biomimetic

## Abstract

Fluorescence probe is one of the most powerful tools for cellular imaging. Here, three phospholipid-mimicking fluorescent probes (FP1–FP3) comprising fluorescein and two lipophilic groups of saturated and/or unsaturated C18 fatty acids were synthesized, and their optical properties were investigated. Like in biological phospholipids, the fluorescein group acts as a hydrophilic polar headgroup and the lipid groups act as hydrophobic non-polar tail groups. Laser confocal microscope images illustrated that FP3, which contains both saturated and unsaturated lipid tails, showed great uptake into the canine adipose-derived mesenchymal stem cells.

## 1 Introduction

Living cell has a plasma membrane that guards the cell nucleus and cytoplasm from the external environment and controls material flow. It is essential that fluorescent probes have membrane affinity to penetrate or accumulate inside of it because the cellular membrane regulates the uptake of extracellular materials inside of the cells. Cell membranes are comprised of phospholipids, cholesterols, and proteins (Yèagle, 1989; Edidin, 2003). The main components are the phospholipids, which comprise an electrically charged polar headgroup and lipophilic fatty acid tails, causing them to form unique assembly structures called lipid bilayers in aqueous media (Daleke, 2003). Cholesterols contained within the bilayer provide membrane flexibility (Brown and London, 1998; Brown and Goldstein, 1999), while proteins loaded on the cell membranes constitute approximately 50 wt% of the entire cell membrane weight. The proteins perform a variety of functions such as regulating material exchange and receiving intercellular signals (von Heijne, 1981). The membrane is the first barrier of cells; therefore, its status is closely related to cell health (Ng et al., 2012).

Several imaging methods have been developed for cell analysis (Chen et al., 2008; García-Sáez and Schwille, 2010; Mohrig et al., 2010; Zhao et al., 2014; Ogiso et al., 2015). Among these, cell imaging using fluorescent probes is useful because of its rapidity, simplicity, and efficiency (Niu et al., 2014; Xu et al., 2014; Lin et al., 2015; Lyu and Pu, 2017; Yan et al., 2017; Wu et al., 2019). In general, fluorescent probes for cell imaging are designed by integrating a cell targeting unit with a fluorophore (Guo et al., 2014; Umezawa et al., 2017; Gao et al., 2019; Xu et al., 2019). Notably, phospholipid-based probes show great affinity to cell membranes owing to their natural similarity to the cell membrane structure. To achieve effective cellular imaging, probes structurally similar with phospholipids have been extensively studied in last two decades. It was reported that a probe containing lipophilic groups effectively penetrates cell membranes. (Honig and Hume, 1989; Yan et al., 2008). Several groups reported novel cellular probes by the introduction of hydrophilic groups (Heek et al., 2013; Zhang et al., 2014; Kreder et al., 2015; Collot et al., 2019b). One of notable developments of probes is oxazolopyridine-based fluorophore by Wang et al. (2021). Oxazolopyridine, which contain both quaternary ammonium groups, show affinity to the outer membrane layer. In addition, simple lipid tail attached on base moiety increases stability of fluorophore when it is merged into inner membrane layer. Cyanine-based fluorescent probes, known as MemBright, have been developed for advanced cellular imaging and neuroscience applications (Collot et al., 2019a). The MemBright fluorophores form aggregates that cause aggregation-caused quenching in aqueous media, thereby minimizing background fluorescence. To balance the lipophilic and hydrophilic properties of probes for effective cell imaging, many synthetic methods have been developed. (Kato et al., 2004; Teramura et al., 2008; Kamitani et al., 2009; Teramura and Iwata, 2010). In addition, various chemical substances for targeting cells have recently been reported. Examples include N-heterocyclic small molecules (Li et al., 2023; Wang et al., 2023), benzothiadiazoles (Neto et al., 2022), and AIE molecules (Xiao et al., 2023).

We aimed at phospholipid-mimicking fluorescent probes for improving permeability into cell membranes. Herein, we report on the synthesis and optical properties of three phospholipid-mimicking fluorescent probes, **FP1**–**FP3**, which comprise fluorescein as a fluorescent body and saturated and/or unsaturated C18 fatty acids as lipophilic tails.

## 2 Materials and methods

### 2.1 General methods

All chemicals were purchased from commercial sources and used without further purification. ^1^H NMR spectra were recorded at 298 K on a 500 MHz JEOL JNM–ECZ 500R/S1 spectrometer. ^1^H NMR data are reported as follows: s: singlet, d: doublet, t: triplet, m: multiplet. Chemical shifts in the ^1^H NMR spectra are reported in parts per million (ppm) compared to a tetramethylsilane (0 ppm) standard. Matrix-assisted laser desorption ionization mass spectrometry (MALDI-TOF-MS) was conducted on a MALDI TOF Voyager DE-STR (Applied Biosystems, USA) mass spectrometer. Thin layer chromatography was performed using silica gel 60 F254 plates. Ultraviolet–visible (UV-vis) absorption spectra were measured with a UV-vis spectrophotometer (Jasco V-670). Fluorescence emission spectra were collected using a Hitachi F-7000 fluorescence spectrophotometer with excitation and emission slit widths of 5 nm. Column chromatography was performed using Merck silica gel (230–400 mesh).

### 2.2 Synthesis

#### 2.2.1 Synthesis of P1

3,5-Dihydroxybenzaldehyde (1 g, 7.2 mmol) and potassium carbonate (4 g, 28.9 mmol) were added to 100 mL of dimethylformamide (DMF) and stirred at 65 °C for 30 min. Stearoyl chloride (4.5 g, 14.8 mmol) was added dropwise under a nitrogen atmosphere. Subsequently, the mixture was slowly heated to 90°C and stirred for 12 h, after which the crude product was extracted and washed three times with ether and water. The solvent was then evaporated under reduced pressure, and the remaining extracted products were purified by silica gel column chromatography with ethyl acetate/hexane (1/10, v/v) as the eluent to obtain 3.5 g of pure product. Yield: 72%; ^1^H-NMR (500 MHz, CDCl_3_): δ 9.95 (s, 1H), 7.49 (d, *J* = 1.1 Hz, 2H), 7.17 (s, 1H), 2.56 (t, *J* = 7.7 Hz, 4H), 1.71–1.77 (m, 4H), 1.25–1.42 (m, 56H), 0.87 (t, *J* = 6.6 Hz, 6H); MALDI-TOF-MS: *m*/*z* 670.65 (calcd. for [M + H]^+^: 671.04).

#### 2.2.2 Synthesis of P2

3,5-Dihydroxybenzaldehyde (1 g, 7.2 mmol) and potassium carbonate (4 g, 28.9 mmol) were added to 100 mL of DMF and stirred at 65 °C for 30 min. Oleoyl chloride (4.5 g, 14.8 mmol) was added dropwise under a nitrogen atmosphere. Subsequently, the mixture was slowly heated to 90°C and stirred for 12 h, after which the crude product was extracted and washed three times with ether and water. The solvent was then evaporated under reduced pressure, and the remaining extracted products were purified by silica gel column chromatography with ethyl acetate/hexane (1/10, v/v) as the eluent to obtain 3.8 g of pure product. Yield: 80%; ^1^H-NMR (500 MHz, CDCl_3_): δ 9.95 (s, 1H), 7.49 (d, *J* = 2.3 Hz, 2H), 7.17 (d, *J* = 2.3 Hz, 1H), 5.32–5.38 (m, 4H), 2.56 (t, *J* = 7.4 Hz, 4H), 2.00 (q, *J* = 6.1 Hz, 8H), 1.71–1.77 (m, 4H), 1.26–1.42 (m, 40H), 0.87 (t, *J* = 6.9 Hz, 6H); MALDI-TOF-MS: *m*/*z* 666.68 (calcd. for [M + H]^+^: 667.01).

#### 2.2.3 Synthesis of P3

3,5-Dihydroxybenzaldehyde (3 g, 21.6 mmol) and potassium carbonate (12 g, 86.7 mmol) were added to 300 mL of DMF and stirred at 65°C for 30 min. Then, oleoyl chloride (6.5 g, 21.6 mmol) was added dropwise under a nitrogen atmosphere, and the mixture was slowly heated to 90°C and stirred for 12 h. The crude product was then extracted and washed three times with ether and water. Subsequently, the solvent was evaporated under reduced pressure, and the remaining extracted products were purified by silica gel column chromatography with ethyl acetate/hexane (1/5, v/v) as the eluent to obtain 3.8 g of pure product. Yield: 60%; ^1^H-NMR (500 MHz, CDCl_3_) δ 9.87 (d, *J* = 5.2 Hz, 1H), 7.16 (t, *J* = 1.7 Hz, 2H), 6.86 (d, *J* = 2.3 Hz, 1H), 5.32–5.38 (m, 2H), 2.56–2.59 (m, 2H), 2.00 (q, *J* = 6.7 Hz, 4H), 1.72–1.78 (m, 2H), 1.26–1.42 (m, 20H), 0.87 (t, *J* = 6.9 Hz, 3H); MALDI-TOF-MS: m/z 402.38 (calcd. for [M + H]^+^: 402.57).

#### 2.2.4 Synthesis of P4

**P3** (3 g, 7.4 mmol) and potassium carbonate (4 g, 28.9 mmol) were added to 100 mL of DMF and stirred at 65°C for 30 min. Stearoyl chloride (4.5 g, 14.8 mmol) was added dropwise under a nitrogen atmosphere. The mixture was slowly heated to 90°C and stirred for 12 h, after which the crude product was extracted and washed three times with ether and water. The solvent was then evaporated under reduced pressure, and the remaining extracted products were purified by silica gel column chromatography with ethyl acetate/hexane (1/10, v/v) as the eluent to obtain 3.7 g of pure product. Yield: 77%; ^1^H-NMR (500 MHz, CDCl_3_): δ 9.95 (s, 1H), 7.49 (d, *J* = 2.3 Hz, 2H), 7.17 (t, *J* = 2.0 Hz, 1H), 5.34 (m, 2H), 2.56 (t, *J* = 7.4 Hz, 4H), 2.01 (t, *J* = 6.3 Hz, 4H), 1.74 (t, *J* = 7.2 Hz, 4H), 1.25–1.41 (m, 48H), 0.87 (t, *J* = 6.9 Hz, 6H); MALDI-TOF-MS: *m*/*z* 669.05 (calcd. for [M + H]^+^: 669.03).

#### 2.2.5 General procedure for the synthesis of probes FP1–FP3

5-Aminofluorescein (0.25 g, 0.72 mmol) was dissolved with **P1**, **P2**, or **P4** (0.72 mmol) in ethyl alcohol (EtOH; 20 mL). The reaction mixture was left for 6 h at reflux. After completion of the reaction, the solvent was evaporated under reduced pressure. The remaining products were purified by silica gel column chromatography with methyl alcohol (MeOH)/CHCl_3_ (1:9, v/v) as the eluent to obtain the final products **FP1**, **FP2**, and **FP3** with yields of 40%, 42%, and 50%, respectively.

##### 2.2.5.1 Probe FP1

^1^H-NMR (500 MHz, DMSO-D_6_): δ 10.07 (s, 1H), 8.78 (s, 1H), 8.00 (d, *J* = 8.6 Hz, 2H), 7.77 (d, *J* = 1.7 Hz, 1H), 7.62 (d, *J* = 8.0 Hz, 1H), 7.27 (d, *J* = 8.0 Hz, 2H), 6.49–6.65 (m, 6H), 2.54–2.59 (m, 6H), 1.93 (t, *J* = 7.4 Hz, 4H), 1.61 (q, 4H), 1.11–1.31 (m, 52H), 0.78–0.81 (m, 6H); MALDI-TOF-MS: *m*/*z* 1000.47 (calcd. for [M + H]^+^: 1000.35).

##### 2.2.5.2 Probe FP2

^1^H-NMR (500 MHz, DMSO-D_6_): δ 10.11 (s, 1H), 8.79 (s, 1H), 7.79 (s, 1H), 7.60–7.62 (m, 2H), 7.53 (s, 1H), 7.28 (d, *J* = 8.0 Hz, 1H), 7.15 (s, 1H), 6.51–6.65 (m, 6H), 5.27–5.30 (m, 4H), 1.94 (t, *J* = 6.5 Hz, 6H), 1.61 (q, *J* = 7.3 Hz, 4H), 1.08–1.26 (m, 48H), 0.70–0.81 (m, 6H); MALDI-TOF-MS: *m*/*z* 996.47 (calcd. for [M + H]^+^: 996.32).

##### 2.2.5.3 Probe FP3

^1^H-NMR (500 MHz, DMSO-D_6_): δ 10.11 (s, 1H), 8.79 (s, 1H), 7.79 (s, 1H), 7.60–7.64 (m, 2H), 7.54 (s, 1H), 7.28 (d, *J* = 8.6 Hz, 1H), 7.15 (s, 1H), 6.49–6.65 (m, 6H), 5.27–5.32 (m, 2H), 2.57 (m, 4H) 1.93 (t, *J* = 6.6 Hz, 4H), 1.61 (q, *J* = 7.3 Hz, 4H), 1.11–1.31 (m, 48H), 0.78–0.81 (m, 6H); MALDI-TOF-MS: *m*/*z* 998.48 (calcd. for [M + H]^+^: 998.33).

### 2.2.6 Preparation of test solution of probes FP1–FP3

A test solution of 10 μM was prepared by diluting a probe solution of 1 mM by 100 times. The dilute solvent was dimethyl sulfoxide.

### 2.2.7 Cell culture

Canine adipose-derived mesenchymal stem cells were incubated in Dulbecco’s modified Eagle’s medium (DMEM) F-12 solution at 37 °C in a 5% CO_2_ atmosphere. For confocal microscopy, cells were plated in 16-well plates (1.5 × 10^4^ cells per well in 200 μL DMEM F-12).

### 2.2.8 Cell toxicity assay

The cytotoxicity of dyes **FP1**–**FP3** was determined by MTT cell proliferation assay (Abcam). Owing to the intracellular reduction of tetrazolium salt to formazan in viable cells, viability can be measured by formazan detection at 595 nm. Each well of a 96-well plate was seeded with 0.75 × 10^4^ canine adipose-derived mesenchymal stem cells in DMEM F-12 at 37°C. After 24 h, the medium was removed, and the cells were treated with a medium containing probes **FP1**–**FP3** (final concentrations of 1, 5, 10, 15, and 20 μM) and incubated for 12 h. For each sample group, 5 wells were prepared. In addition, each plate contained three control wells (cells with untreated DMEM F-12). After incubation, 15 μL of MTT solution was added to each well and incubated for 4 h at 37 °C. Subsequently, 100 μL of the solubilization/stop solution was added to each well. After further incubation for 24 h, the absorbance at 595 nm was recorded using a 96-well plate reader.

### 2.2.9 Cell confocal imaging

For the laser confocal fluorescence imaging experiments, cells were incubated with probes **FP1**–**FP3** (10 μM) for 30 min. After incubation with probes **FP1**–**FP3**, the incubated cells were washed with phosphate-buffered saline (PBS) three times and then photographed by confocal microscopy. Green channel images (490–550 nm) were obtained upon excitation at 488 nm. All cell images were processed using Zeiss ZEN 3.1 software.

### 2.2.10 Flow cytometry analysis

Cells were cultured to populations of 10^6^ cells each with PBS buffer solution. The prepared cells were stained with probes **FP1**–**FP3** for 4 h, then washed two times with PBS and transferred to a Falcon 5 mL test tube for analysis using a BD FACSCalibur flow cytometer. The results were analyzed with FCS Express flow cytometry software. The cellular membrane-bound probes were quantified based on the relative intensity of the fluorescence signal to the average mean fluorescence intensity of the control group.

## 3 Results

### 3.1 Design and synthesis

**SCHEME 1:**
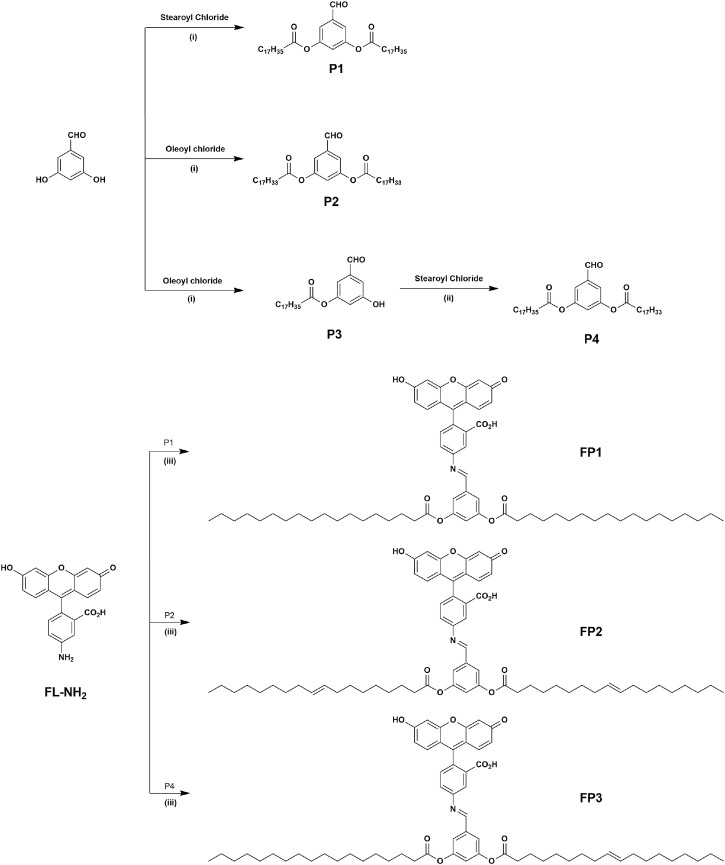
Synthesis of products P1–P4 and probes FP1–FP3: (i) K_2_CO_3_, DMF, 65°C, 8 h; (ii) K_2_CO_3_, DMF, 90°C, 12 h; (iii) EtOH, reflux for 6 h.

**FP1-FP3** probes were designed to have similar structures with typical phospholipid molecules. In order to make phospholipid-like fluorescent probes, fluorescein is selected to be hydrophilic head group, for its biocompatibility and high hydrophilicity. Furthermore, probes were attached with artificial lipid tails moiety instead of cell-originated lipid tails. Advantages from this method is not only easy synthesis, but also easy to change which tails would be attached between saturated and/or unsaturated lipid tails. The synthetic routes of probes **FP1**–**FP3** are shown in Scheme 1. **P1**, **P2**, and **P4** are prepared by the acylation of saturated and unsaturated acyl chlorides, with yields of 70%–80%. Probes **FP1**, **FP2**, and **FP3** were synthesized by the condensation of 5-aminofluorescein with **P1**, **P2**, and **P4**, respectively, with yields of 40%, 42%, and 50%, respectively. **FP1**–**FP3** were fully characterized using ^1^H-NMR spectroscopy and MALDI-TOF MS.

### 3.2 Photophysical properties

**FIGURE 1:**
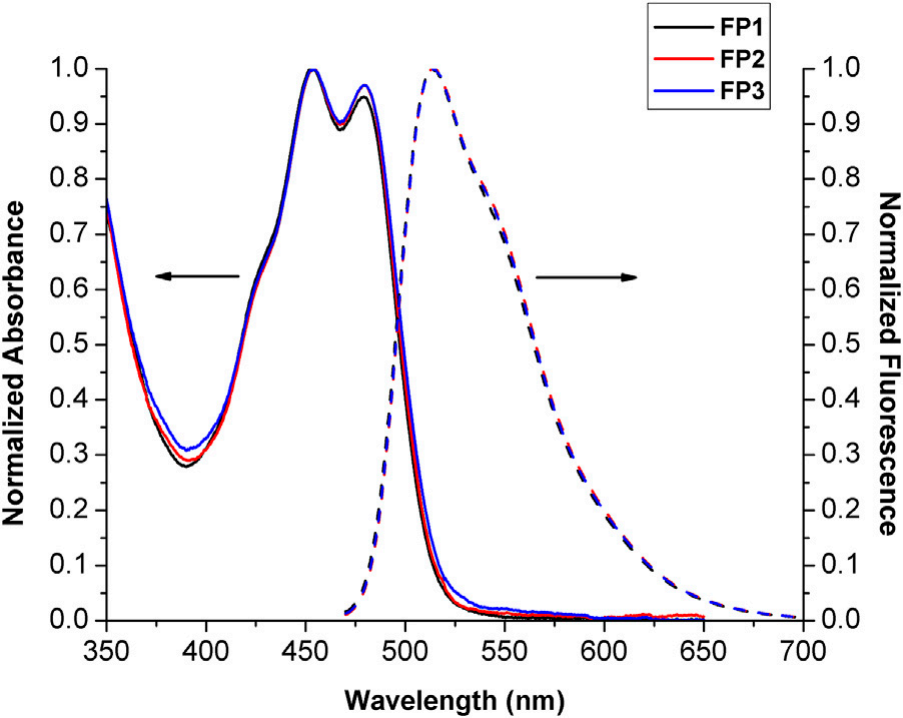
Normalized absorbance and emission spectra (*λ*_ex_ = 452 nm) of probes FP1–FP3 (10 μM) in MeOH. The absorbance and emission spectra are drawn with solid and broken lines, respectively.

The optical properties of probes **FP1**–**FP3** were investigated by measuring their absorption and emission spectra in MeOH at a concentration of 10 μM. The results are shown in Figure 1. Because the structure of the fluorescein group is not directly modified by the synthetic procedure, the absorbance spectra of **FP1**–**FP3** were almost identical, with two absorption peaks at 452 and 480 nm. The fluorescence emission spectra also showed a minimal difference, with a single peak at 513 nm. Fluorescence quantum yields (Φ_f_) for **FP1**, **FP2**, and **FP3** were calculated to be 0.36, 0.34, and 0.38, respectively, compared to the standard (fluorescein, *Φ*_f_ = 0.91 in MeOH). (Magde et al., 2002; Levitus, 2020). These results demonstrate that the biomimetic phospholipid tails do not affect the photophysical properties of the fluorophore to which they are attached.

### 3.3 Cell toxicity and fluorescence imaging of living cells

**FIGURE 2:**
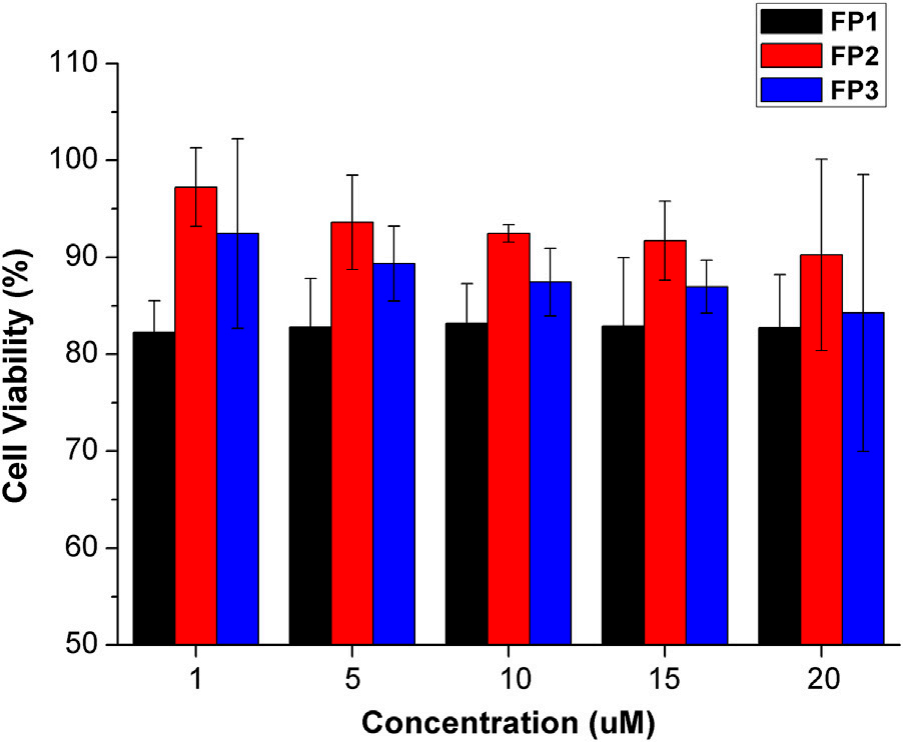
Viability of cells treated with probes FP1–FP3 at concentrations of 1, 5, 10, 15, 20 μM. Cell viability was assessed by MTT assay. Data are the mean ± S.D. of five independent experiments.

**FIGURE 3:**
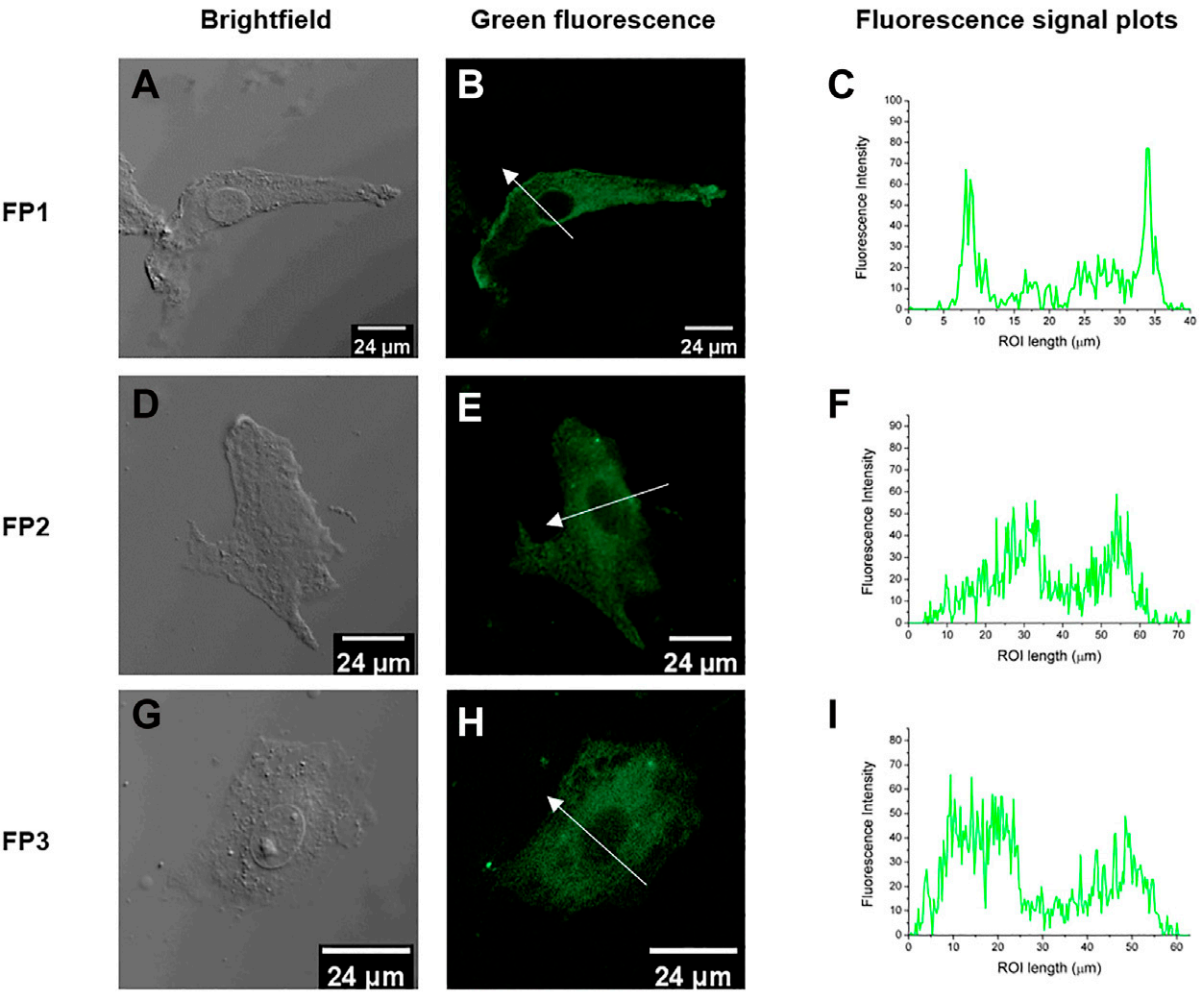
Fluorescence confocal images of living canine adipose-derived mesenchymal stem cells treated with probes FP1, FP2, and FP3 (10 μM); **(A, D, G)** Bright field images; **(B, E, H)** confocal images (green channel) of FP1–FP3, respectively; **(C,F, I)** Fluorescence signal plots in the regions of interest (ROI) (white arrows in **(B, E, H)**. Green channel emission was collected at 490–550 nm, upon excitation at 488 nm.

The probes qualified as hypotoxic, as shown in Figure 2. Thus, cell imaging experiments were carried out in living canine adipose mesenchymal stem cells to study the organelle-targeting behavior of the probes. Stem cells were stained using probes **FP1**–**FP3** (10 μM; green channel) and cultured in an incubator at 37°C for 30 min. The fluorescence images were recorded by confocal laser scanning microscopy (Figure 3). The imaging results showed that all three fluorescent probes reside inside of the cells. In specific, **FP1** appeared to be weakly localized in the thickest part of the cell membrane, while **FP2** and **FP3** are almost internalized in cell cytoplasm.

### 3.4 Flow cytometry analysis

**FIGURE 4:**
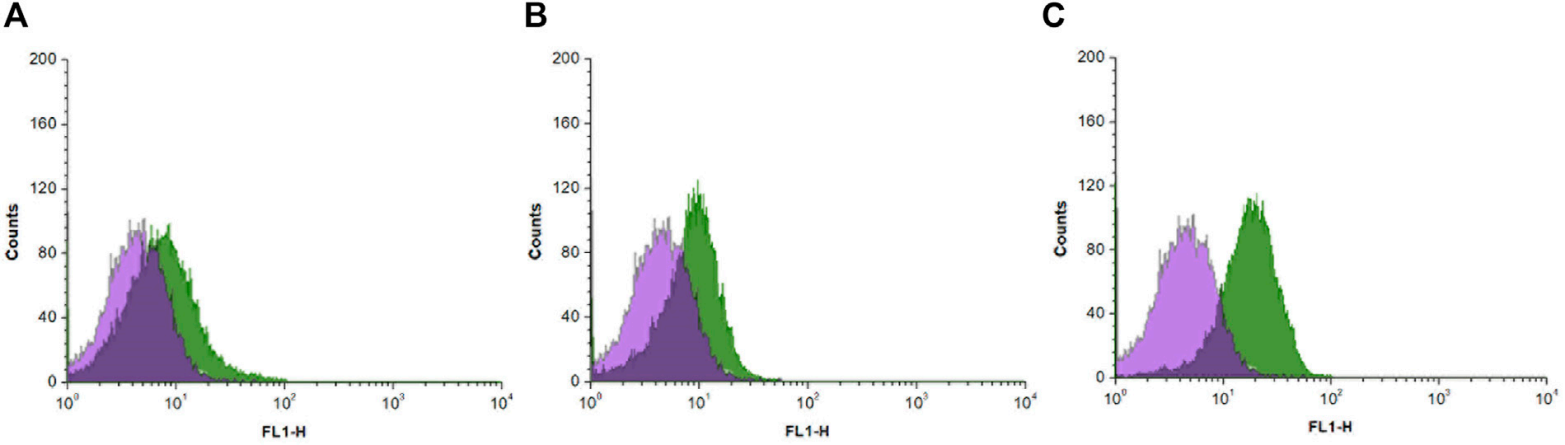
Histograms of flow cytometry analysis results of canine adipose-derived mesenchymal stem cells treated with **(A)** FP1 **(B)** FP2, and **(C)** FP3, showing the fluorescence intensity of the probes in cells.

Flow cytometry is an analytical technique that utilizes light to count and profile cells in a heterogenous fluid mixture. Stem cells were treated with probes **FP1**–**FP3** and analyzed by flow cytometry. There was an overall increase in the fluorescence intensity after treatment with all three probes (Figure 4). **FP1** and **FP2** showed mean increases of 81.4% and 80.8%, respectively. Surprisingly, **FP3** showed a more significant increase of 294%.

## 4 Discussion

In summary, a series of fluorescein-based cell staining probes based on a biomimetic phospholipid-like structure were designed and synthesized. The basic structure of a cell membrane consists of a bilayer scaffold of phospholipids with hydrophilic headgroups and hydrophobic alkyl chain tails. For biological applications, fluorescent probes need to have good biocompatibility. Fluorescein is hydrophilic, which prevents it from aggregating in microcellular environments, and its biocompatibility is well studied and accepted. Probes **FP1**–**FP3** had almost identical optical properties to those of the parent molecule, fluorescein. They also had low cytotoxicity toward canine adipose mesenchymal stem cells. In confocal laser scanning microscopy results, **FP1-FP3** achieved good cell staining ability. Cells washed after the staining procedure showed green fluorescence under the excitation light, and the intensity profile along the cross-sectional ROI of **FP1-FP3** clearly demonstrated that the probe penetrated into the cells and accumulated inside the cytoplasm. Flow cytometry has shown that probe **FP3**, which contains both saturated and unsaturated C18 alkyl chains, is having exquisite internalization ability towards the cell cytoplasm through the cell membrane. These results might provide a better understanding of the design of fluorescent molecules for bioimaging. That is, unsaturated alkyl chains are relatively rigid, but long saturated alkyl chains are flexible (Subczynski et al., 1993; Yamada et al., 2022). Like phospholipid, **FP3** molecules with both rigid and flexible tails are favorable structures for cytoplasmic internalization.

## Data Availability

The original contributions presented in the study are included in the article/Supplementary Materials, further inquiries can be directed to the corresponding authors.
