# Supplementary material for: Biomimetic lipid–fluorescein probe for cellular bioimaging
Source: Front Chem. 2023 Apr 21;11:1151526. doi: 10.3389/fchem.2023.1151526 (PMC10160471; doi:10.3389/fchem.2023.1151526)
Supplement: Supplementary file 1 [file DataSheet1.docx]

Supplementary Material

Biomimetic lipid–fluorescein probe for cellular bioimaging

Hyungkyu Moon^1^, Tania Sultana^3^, JeongIk Lee^4*^, Jungrim Huh^2^, Hae Dong Lee^1^, Myung-Seok Choi^1*^

*** Correspondence:** Myung-Seok Choi, mchoi@konkuk.ac.kr; JeongIk Lee, jeongik@konkuk.ac.kr

**Table of Contents**

1. MALDI-TOF-MS spectra of **P1-P4** and **FP1-3** S2-S8
2. ^1^H-NMR spectra of **P1-P4 and FP1-3** S9-S16

## Supplementary Figures



 **Figure S1.** MALDI-TOF-MS spectrum of **P1**



 **Figure S2.** MALDI-TOF-MS spectrum of **P2**



 **Figure S3.** MALDI-TOF-MS spectrum of **P3**

**

 Figure S4.** MALDI-TOF-MS spectrum of **P4**

**

 Figure S5.** MALDI-TOF-MS spectrum of **FP1**

**

 Figure S6.** MALDI-TOF-MS spectrum of **FP2**

**

 Figure S7.** MALDI-TOF-MS spectrum of **FP3**

**
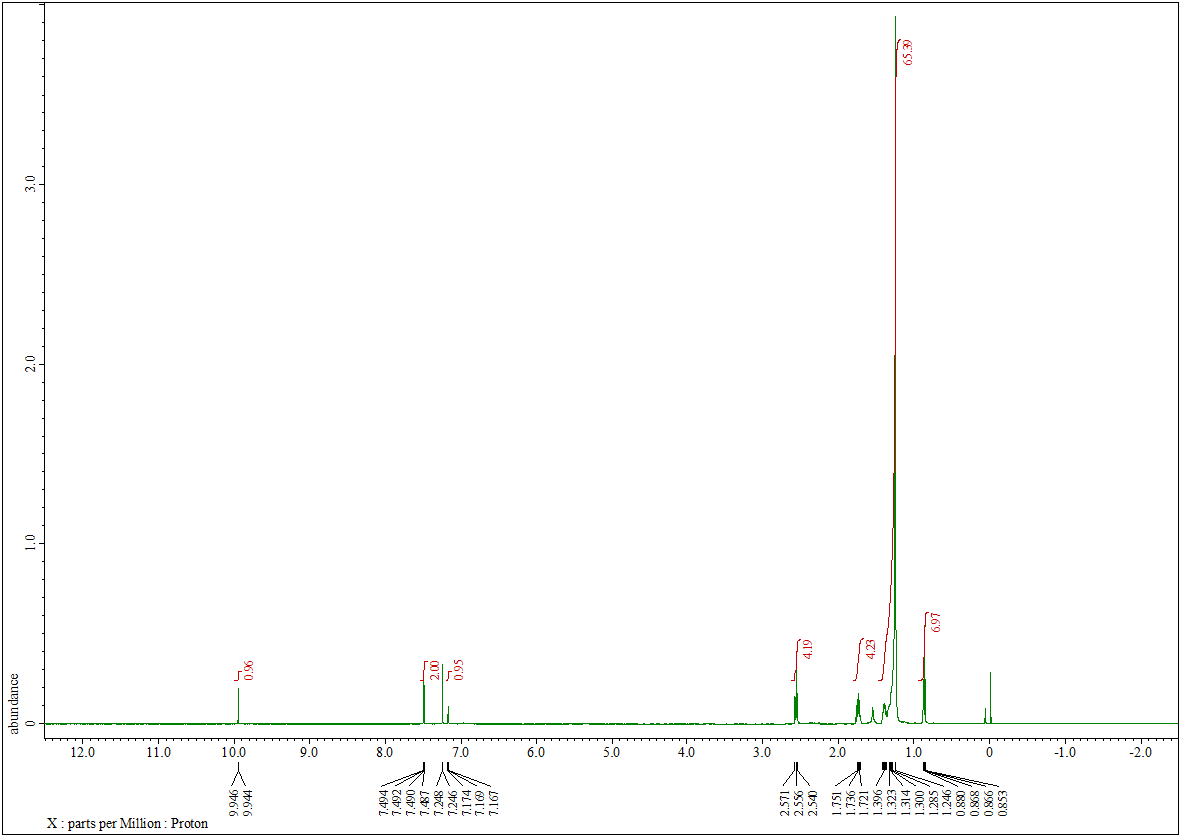
**

**Figure S8.** ^1^H-NMR spectrum of **P1**

**Figure S9.** ^1^H-NMR spectrum of **P2**

**Figure S10.** ^1^H-NMR spectrum of **P3**

**Figure S11.** ^1^H-NMR spectrum of **P4**

**Figure S12.** ^1^H-NMR spectrum of **FP1**

**Figure S13.** ^1^H-NMR spectrum of **FP2**

**Figure S14.** ^1^H-NMR spectrum of **FP3**
